# Management of a human immunodeficiency virus case with discordant antiviral drug resistance profiles in cerebrospinal fluid compared with plasma: a case report

**DOI:** 10.1186/s13256-022-03289-8

**Published:** 2022-02-15

**Authors:** Didi Bang, Jannik Fonager, Isik Somuncu Johansen

**Affiliations:** 1 Department of Clinical Microbiology, Copenhagen University Hospital, Amager and Hvidovre, Copenhagen, Denmark; 2 Virus and Microbiological Special Diagnostics, Statens Serum Institut, Copenhagen, Denmark; 3 Department of infectious Diseases Q, Odense University Hospital, Odense, Denmark

**Keywords:** HIV, AIDS, Cerebrospinal fluid, HIV-associated neurocognitive disorder, Protease inhibitor drug resistance

## Abstract

**Background:**

Human immunodeficiency virus-1-associated neurocognitive disorder is a known complication in individuals treated with antiretroviral therapy. Cerebrospinal fluid escape, which is defined as discordant higher cerebrospinal fluid viremia than plasma, may occur in antiretroviral therapy-experienced individuals. Different cerebrospinal fluid versus plasma mutation patterns have been observed in individuals with cerebrospinal fluid escape.

**Case presentation:**

A 46-year-old adult African male with human immunodeficiency virus-1 infection and acquired immunodeficiency syndrome based on cerebral toxoplasmosis and a chronic hepatitis B virus infection developed cerebrospinal fluid escape. A different human immunodeficiency virus-1 genotypic drug resistance profile was observed in plasma compared with cerebrospinal fluid. Brain biopsy and cerebral magnetic resonance imaging indicated the development of human immunodeficiency virus encephalopathy. A discordant protease inhibitor mutation/wild-type T74PT in plasma but not in cerebrospinal fluid indicated poor central nervous system penetration due to the selective pressure of drug therapy. An intensified antiretroviral therapy regimen including dolutegravir with good central nervous system penetration improved conditions.

**Conclusions:**

This case shows the importance of measuring human immunodeficiency virus drug resistance in cerebrospinal fluid, which might differ from resistance detected in plasma samples and target effective antiretroviral therapy treatment accordingly.

## Background

The management of human immunodeficiency virus (HIV) drug resistance is challenging [1]. Few studies describe differences in genotypic resistance profiles in plasma compared with other compartments such as cerebrospinal fluid (CSF). The central nervous system (CNS) is an important escape sanctuary for HIV [2]. Poor compliance and co-infections with other pathogens may complicate treatment.

## Case presentation

**Fig. 1:**
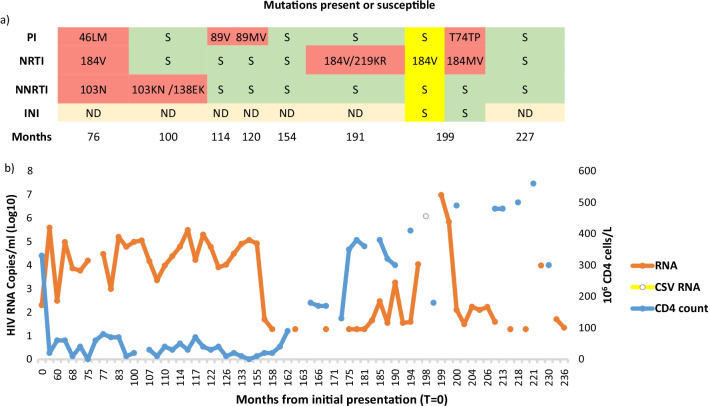
Resistance mutation development, antiretroviral therapy, human immunodeficiency virus-1 RNA viral load, and cluster of differentiation 4 cell count over time. **a** human immunodeficiency virus resistance mutation patterns are shown (red background), susceptible (green background), cerebrospinal fluid findings (yellow background), and time in months from the initial presentation (*T* = 0). **b** Plasma and cerebrospinal human immunodeficiency virus-1 RNA viral load and cluster of differentiation 4 cell count are shown over time. Antiretroviral therapy was commenced with a three-drug combination therapy including two nucleoside(tide) reverse-transcriptase inhibitors (lamivudine, zidovudine), and the non-nucleoside reverse transcriptase EFV 60 months after initial presentation. The non-nucleoside reverse transcriptase was discontinued and a protease inhibitor was added (month 75). Therapy was intensified to a four-drug regimen (month 169) with the inclusion of the INI raltegravir, and later substituted by the more potent dolutegravir (month 227). The different nucleoside(tide) reverse-transcriptase inhibitors used for treatment included lamivudine, zidovudine, tenofovir, abacavir, and emtricitabine). Protease inhibitors used were nelfinavir, lopinavir/ritonavir, atazanavir, and darunavir

A 46-year-old male from Togo with HIV-1 and acquired immunodeficiency syndrome (AIDS) presented with confusion despite receiving antiretroviral therapy (ART) for more than a decade. AIDS was due to previous cerebral toxoplasmosis complicated with epilepsy and a chronic hepatitis B virus co-infection. ART was initiated at a baseline cluster of differentiation 4 (CD4) count of 330 cells/mm^3^. During the first years, ART consisted of two nucleoside(tide) reverse-transcriptase inhibitors (NRTIs) and a non-nucleoside reverse transcriptase (NNRTI). The HIV-1 *pol* gene genotypic resistance analysis showed development of the NRTI M184V mutation, and NNRTI K103N and E138EK mutations in plasma, respectively. Poor compliance, increasing viral loads, falling CD4 cell count levels, and mutation development led to therapy changes. The lowest nadir CD4 count was zero cells/mm^3^. The NRTI M184V mutation results in high-level *in vitro *resistance to lamivudine (3TC) [3]. As the mutation increases susceptibility to zidovudine (AZT) and tenofovir (TDF), combined treatment with 3TC was continued. The nonpolymorphic NNRTI K103N mutation causes high-level resistance to efavirenz (EFV), and E138K mutation causes potential low-level cross-resistance to EFV, which was discontinued together with 3TC. Therapy now consisted of two to four of the newer potent NRTIs with addition of a protease inhibitor (PI) first alone and later boosted (Fig. 1).

During subsequent years, the plasma resistance pattern reverted to susceptible for the NRTI/NNRTIs; however the PI mutation L89V appeared, only later to revert to fully susceptible again. The PI L89V mutation may contribute to reduced PI susceptibility. Strengthened therapy included an integrase inhibitor (INI).

After more than a decade on ART therapy, HIV RNA levels and CD4 cell counts deteriorated and the condition was complicated by unspecific neurological symptoms and convulsions. The CSF cell count was increased with 99 (>5) cells per mm^3^ (98% mononuclear), with an elevated protein level (1.96, 0.4–0.7 g/L), no erythrocytes present, and a normal glucose level (2.5 mmol/l). Cerebrospinal fluid was negative for bacterial culture, syphilis, toxoplasmosis, *Mycoplasma pneumoniae*, and polymerase chain reaction (PCR) diagnostics for John Cunningham (JC) polyomavirus, enterovirus, herpes simplex virus, varicella zoster virus, and cytomegalovirus. The CSF Epstein–Barr virus (EBV) level was 11,871 copies per ml. The HIV viral load in CSF and plasma were 1.2 million and 10,874 HIV RNA copies per ml, respectively. Cerebral magnetic resonance imaging (MRI) showed toxoplasmosis sequelae and unspecific changes in the white matter. A brain biopsy confirmed a diagnosis of HIV encephalopathy with cerebral toxoplasmosis sequelae.

At the point of CSF escape diagnosis, the CD4 cell count was 180 cells/mm^3^, and both plasma and CSF harbored the same NRTI M184V mutation. However, discordantly, the accessory nonpolymorphic PI T74TP mutation mixed with wild type appeared only in the plasma but not CSF. Intensified therapy included changing raltegravir with the more potent integrase inhibitor dolutegravir, which resulted in a marked improvement of CD4 cell counts and suppression of viral levels.

## Discussion and conclusion

Cerebral MRI and histopathology indicated HIV encephalopathy. The blood–brain barrier may hinder the passage of ART into the brain parenchyma. Reduced penetration achieving lower CNS concentrations leads to virus compartmentalization and selection of drug-resistant mutants [4]. The discordant finding of the PI T74TP mutation/wild type in the plasma but not CSF indicates a selective drug pressure in plasma and not in the CSF caused by insufficient CNS drug penetration. The mutation occurs primarily in patients who have received multiples PIs, and may cause potential low-level resistance. The CNS distribution of ART may be poor, and the CSF concentrations may fall below the drug concentrations needed to inhibit wild-type virus [5]. Despite ART, about half of all HIV-infected individuals experience cognitive impairment [5]. Our case findings were consistent with characteristics frequently observed with CSF escape. Cerebrospinal fluid escape is seen after more than 15 years of HIV infection, previous low-level viremia, and the presence of M184V/I mutations in the CSF [6]. Antiretroviral therapy may have a greater effect on HIV-RNA levels in the CSF compared with plasma, even in the presence of drug resistance. The CNS Penetration Effectiveness (CPE) score is designed to rank the effectiveness of each drug. The score is high for boosted PIs, which are considered to be CNS penetrating [2, 7]. Boosted PIs may reduce the CSF HIV-RNA level but do not consistently achieve *in vitro* 50% inhibitory concentrations in the CSF [7]. PI regimens have been associated with threefold higher odds of CSF escape compared with other ART regimens and neurological symptoms [7]. A large prospective study has shown that reduced CNS ART bioavailability may predispose to CSF escape in patients with MI84V/I mutations, as found in this case [7].

Our case had CSF viral escape with a CSF HIV-RNA level more than 100 times higher than that observed in plasma. This could lead to speculation on the adherence level. Although the T74P mutation is described as nonpolymorphic, the T74TP mutation was mixed with wild type, which has sporadically been reported. This partial reversion to wild type might be explained by prolonged periods of nonadherence. Intensified ART with good CNS penetration drugs, including the integrase inhibitor dolutegravir, which has the highest CPE score, markedly improved neurological symptoms and regression of cerebral findings on repeated MRI scans. Although less neurotropic, the findings of elevated EBV in the CNS may also have contributed to the HIV encephalopathy observed. This case shows the importance of measuring HIV viral load and drug resistance in CSF fluid in patients with neurological symptoms, allowing for effective therapy adjustments and improved management of HIV-associated neurocognitive disorders.

Complete control of HIV replication in the CSF as well as in plasma is necessary.

## Data Availability

All data generated or analyzed during this study are included in this published article.

## Abbreviations

ART: Antiretroviral therapy
CNS: Central nervous system
CPE: Central nervous system penetration effectiveness
CSF: Cerebrospinal fluid
EBV: Epstein–Barr-virus
MRI: Magnetic resonance imaging
HIV: Human immunodeficiency virus
INI: Integrase inhibitor
NRTI: Nucleoside(tide) reverse transcriptase inhibitor
NNRTI: Non-nucleoside reverse-transcriptase inhibitor
PI: Protease inhibitor
S: Susceptible
AZT: Zidovudine
3TC: Lamivudine
ABC: Abacavir
TDF: Tenofovir disoproxil
FTC: Emtricitabine
EFV: Efavirenz
NFV: Nelfinavir
LPVr: Lopinavir/ritonavir
ATV: Atazanavir
DRV: Darunavir
RAL: Raltegravir
DTG: Dolutegravir

## References

[1] Clavel F, Allan J, Hance AJ. HIV drug resistance. N Engl J Med. 2004;350:1023–35. 10.1056/NEJMra025195 14999114

[2] Soulie C, Grudé M, Descamps D, Amiel C, Morand-Joubert L, Raymond S, *et al*. Antiretroviral-treated HIV-1 patients can harbour resistant viruses in CSF despite an undetectable viral load in plasma. J Antimicrob Chemother. 2017;72:2351–4. 10.1093/jac/dkx128 28472307

[3] Rhee S, Gonzales MJ, Kantor R, Betts BJ, Ravela J, Shafer RW. Human immunodeficiency virus reverse transcriptase and protease sequence database. Nucleic Acids Res. 2003;31:298–303. 10.1093/nar/gkg100 PMC165547 12520007

[4] Nightingale S, Geretti AM, Beloukas A, Fisher M, Winston A, Else L, *et al*. Discordant CSF/plasma HIV-1 RNA in patients with unexplained low-level viraemia. J Neurovirol. 2016;22:852–60. 10.1007/s13365-016-0448-1 PMC5127885 27194435

[5] Nightingale S, Winston A, Letendre S, Michael BD, Mcarthur JC, Khoo S, *et al*. Controversies in HIV-associated neurocognitive disorders. Lancet Neurol. 2014;13:1139–51. 10.1016/S1474-4422(14)70137-1 PMC4313542 25316020

[6] Mukerji SS, Misra V, Lorenz D, Cervantes-Arslanian AM, Lyons J, Chalkias S, *et al*. Temporal patterns and drug resistance in CSF viral escape among ART-experienced HIV-1 infected adults. J Acquir Immune Defic Syndr. 2017;75:246–55. 10.1097/QAI.0000000000001362 PMC5452976 28328546

[7] Mukerji SS, Misra V, Lorenz DR, Uno H, Morgello S, Franklin D, *et al*. Impact of antiretroviral regimens on cerebrospinal fluid viral escape in a prospective multicohort study of antiretroviral therapy-experienced human immunodeficiency virus-1-infected adults in the United States. Clin Infect Dis. 2018;67:1182–90. 10.1093/cid/ciy267 PMC6160603 29617912

